# Curriculum Innovations: A Comprehensive Teleneurology Curriculum for Neurology Trainees

**DOI:** 10.1212/NE9.0000000000200084

**Published:** 2023-08-03

**Authors:** Steve C. Han, Rebecca S. Stainman, Neil A. Busis, Scott N. Grossman, Sujata P. Thawani, Arielle M. Kurzweil

**Affiliations:** From the Department of Neurology (S.C.H., N.A.B., S.N.G., S.P.T., A.M.K.), New York University Grossman School of Medicine, New York; and Department of Neurology (R.S.S.), Johns Hopkins School of Medicine, Baltimore, MD.

## Abstract

**Introduction and Problem Statement:**

As the role of teleneurology expands, it is important to prepare trainees to perform virtual encounters proficiently.

**Objectives:**

We created a comprehensive multimodality teleneurology curriculum for residents to teach key aspects of telehealth encounters including the virtual examination and skill development across several environments.

**Methods and Curriculum Description:**

We developed and implemented a teleneurology curriculum focused on teaching the virtual neurologic examination, measuring teleneurology competency, and providing opportunities for trainees to perform telehealth encounters in multiple settings. Residents (N = 22) were first surveyed on what methods would be most helpful to learn teleneurology. Trainees observed a faculty member conducting a teleneurology visit with another faculty member playing a patient. Residents then practiced a teleneurology encounter during a 10-minute objective structured clinical examination (OSCE) at a simulation center. After positive feedback from the fall of 2020, we adapted the OSCE to be completely remote in the spring of 2021 for senior residents. Trainees then performed teleneurology visits during their continuity clinics and subspecialty clinic rotations.

**Results and Assessment Data:**

All neurology residents from adult and child neurology and neuropsychiatry programs at New York University Grossman School of Medicine participated in the curriculum. Residents identified a variety of teaching modalities that would help them learn teleneurology: didactics with slides (25%), live demonstration (25%), simulated experience (23%), starting with live patients (23%), and articles/reading material (4%). To date, 68 trainees participated in the OSCE. Trainees who completed on-site and remote simulations reported increased comfort (*p* < 0.05) and interest in teleneurology (*p* < 0.05) and requested more access to simulations during training. Sensorimotor assessment and adequate visualization of the affected limb were identified as areas for improvement.

**Discussion and Lessons Learned:**

Our multimodal 3-year teleneurology curriculum provides opportunities for residents to learn and apply teleneurology. Survey tools helped strengthen the curriculum to optimize educational potential. We implemented a teleneurology simulation with and without the use of a simulation center. We plan to expand our teleneurology clinical and simulation experiences to trainees based on our data and further developments in teleneurology and to track the progress of teleneurology skills as residents advance through training.

## Introduction and Problem Statement

As teleneurology evolves, trainees need to learn how to perform virtual encounters proficiently.^1,2^ The Accreditation Council for Graduate Medical Education permitted the use of telemedicine by residents and fellows with supervision in March 2020.^3^

Frameworks for developing and evaluating telemedicine competency have been promulgated.^4-6^ The American Academy of Neurology Telemedicine Work Group published recommendations for curricular development and trainee teleneurology practice equivalencies to meet patients' needs in 2017. At the beginning of the coronavirus disease 2019 (COVID-19) pandemic, leaders in medical education and virtual care proposed evaluating learners' competencies virtually.^7^ Programs adapted existing objective structured clinical examinations (OSCEs) for telehealth training.^8^ Others created opportunities for residents and medical students to learn or practice teleneurology, including virtual OSCEs for medical students.^9-14^ Despite progress, formalized evidence-based guidelines for teaching teleneurology remain in early stages of development.^15^

Teaching residents proper physical examination techniques is fundamental to their training. In-person examination skills are not necessarily transferrable to virtual settings.^2,16-20^ Programs are increasingly implementing telemedicine curricula for trainees and medical students.^9,11,12,21-23^ The NIH Stroke Scale (NIHSS) and mental status assessments have been adapted for remote use.^24^ Best practices for other elements of the remote neurologic examination are being developed.^16,17,24,25^

## Objectives

There are gaps in understanding:

- How residents perceive teleneurology
- Residents' preferred methods of learning teleneurology
- How on-site or remote simulation-based learning effect residents' interest in and comfort with virtual visits
- Residents' competencies with “webside” manner and the virtual neurologic examination

**Figure 1:**
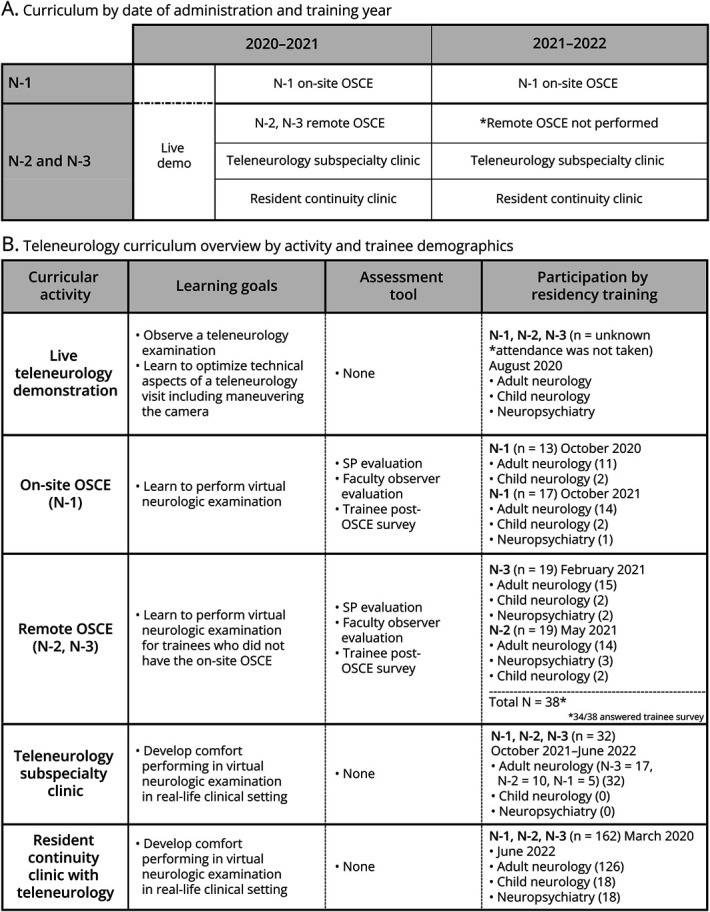
Overview of the Teleneurology Curriculum and the Participants This schematic summarizes the details of the curriculum and the timing of the curricular activities. (A) Exhibits the major teleneurology-related activities at specific training years. (B) Summarizes major learning goals of these teleneurology-related activities, the relevant assessment tools, as well as the participant demographics. OSCE = objective structured clinical examination.

To address these gaps, we developed and implemented a comprehensive teleneurology curriculum for the neurology training programs at the New York University Grossman School of Medicine (NYUGSOM) (Figure 1, A and B). We aimed to teach key aspects of a virtual visit including the virtual neurologic examination, measure trainee comfort/interest in teleneurology, and provide opportunities to implement trainees' skills in multiple environments. Previously, our trainees received no formal education in virtual care nor did they perform teleneurology encounters.

## Methods and Curriculum Description

Our neurology department transitioned to virtual visits on March 19, 2020. We developed a general teleneurology examination. We performed remote visits by adapting existing technological infrastructure developed for virtual urgent care visits.^16^

### Curriculum Participants

All NYUGSOM neurology residents from adult and child neurology and neuropsychiatry programs participated. Participation in surveys was optional. In this study, “neurology residents” encompasses all these trainees. We delineate types of residents when possible. “First-year neurology residents” are trainees who completed the prerequisite 1 year of medicine residency or 2 years of pediatrics and are in their first year of neurology training (Figure 1, A and B). The neuropsychiatry program refers to our double-board neurology and psychiatry residency program.

### Curriculum Evaluation

Using the Kirkpatrick model, we conducted quantitative and qualitative evaluations of our curriculum's ability to fulfill our objectives with a focus on trainee reaction and learning.^26^ A voluntary anonymous precurricular survey assessed residents' perspectives on teleneurology and preferred methods of learning using a 5-point Likert scale, a multioption question, and a free-text response (Kirkpatrick level 1). The pre-OSCE and post-OSCE questions using a 5-point Likert scale assessed changes in the trainees' interest in and comfort with teleneurology after the simulation (Kirkpatrick level 2).

#### Needs Assessment: Precurricular Survey

Prior to the development of the teleneurology curriculum in 2020, all adult and child neurology residents and neuropsychiatry residents were asked to voluntarily complete a 9-question anonymous online survey to assess their perspectives on teleneurology (eTable 1, links.lww.com/NE9/A40). Anonymity, intended to maximize response rate, precluded an exact breakdown of residents by program or year. Seven questions used a 5-point Likert scale (1 = strongly agree, 2 = agree, 3 = neutral, 4 = disagree, and 5 = strongly disagree). One question asked trainees to select up to 2 preferred methods of learning teleneurology from a list of preselected common learning modalities. One question requested a free-text response where residents could reflect on a specific area of teleneurology in which they desired further training. This survey served as a needs assessment for curricular development and was not repeated after the first year of the curriculum.

#### Virtual Visit Demonstration

After reviewing the results of the precurricular survey, we developed a live demonstration of a complete teleneurology encounter including technical and clinical aspects. In August 2020, during a residents' 1-hour noon conference, 2 faculty members demonstrated a 2-way real time audio-video teleneurology encounter. All participated via video conferencing software.

One faculty member role-played a physician while the other faculty member role-played a patient presenting with clinical signs and symptoms suggestive of compressive ulnar neuropathy at the elbow. Trainees on inpatient services observed the session from a conference room. Trainees on outpatient rotations observed the session from devices of their choosing. For the first 30 minutes, the faculty member demonstrated components of a new patient encounter including taking a history and performing a virtual neurologic examination. The remaining 30 minutes were reserved for interactive questions and answers from the audience. The session was recorded and uploaded to a shared online education portal. This recording served as an enduring resource for trainees to review and reinforce teleneurology skills. We also uploaded key teleneurology reading materials for trainees' self-study.

#### Objective Structured Clinical Examinations

Residents, as adult learners, learn best by doing.^27^ In 2020, we developed and piloted an OSCE case that can be completed in a simulation center or via remote connection in collaboration with the New York Simulation Center (NYSIM) medical education faculty. This simulation was incorporated into a preexisting 4-station OSCE on core competencies for first-year trainees in adult neurology, child neurology, and neuropsychiatry double-board residency programs.

A standardized patient (SP) presented with signs and symptoms consistent with meralgia paresthetica. Trainees were instructed to briefly confirm the history, examine the SP, and then discuss their assessment and plan. We selected a condition commonly seen in the outpatient neurology clinic setting that required a focused motor and sensory examination. We chose meralgia paresthetica because it required repositioning of the patient and camera to visualize the affected body part. The SP portrayed a patient with new-onset tingling in the right lateral thigh after starting a security job that required wearing a heavy work belt. The new work schedule made an in-person appointment difficult, so a teleneurology new patient visit was scheduled (eFigure 1, A and B, links.lww.com/NE9/A40).

Neurology faculty and an SP educator trained an SP for 2 hours on how to portray a patient with signs and symptoms consistent with compression of the lateral femoral cutaneous nerve. The SP also learned how to complete an evaluative checklist, which primarily assessed trainees' communication skills (eTable 2, links.lww.com/NE9/A40). This case-specific checklist was developed by a multidisciplinary group of neurology faculty and experienced telemedicine clinicians at NYU and has been validated in other OSCEs.^28,29^ The assessment was shared with trainees after the session.

Residents received verbal feedback from a faculty observer. During the initial implementation of the in-person OSCE, the faculty observer documented free-text feedback regarding the trainees' performance. For subsequent teleneurology OSCEs, neurology faculty used a standardized physical examination checklist developed in collaboration with simulation center personnel (eTable 3, links.lww.com/NE9/A40).^16^

Prior to starting the encounter, trainees were given information detailing the patient's presentation (eFigure 1C, links.lww.com/NE9/A40). They were instructed to confirm or clarify pertinent history; perform a virtual neurologic examination, including the basic neurologic components for each system (they could assume the mental status examination was intact); and briefly discuss the assessment and plan with the patient.

The neurology department covered costs incurred by the NYSIM including finding, recruiting, and training the SP. Our SP spent 7 hours at the simulation center on the day of the simulation and was reimbursed at an hourly rate.

##### On-site OSCE Logistics

The resident and SP sat alone in adjacent simulation center rooms and used a videoconferencing platform (Zoom) for the encounter. The camera, speaker, and microphone were turned on. A faculty member observed through 1-way glass windows with views into the SP and resident rooms and accessed the virtual meeting room via a video window that was not visible to the SP or learner.

Each encounter started with the screen showing only the top half of the SP's face, to mimic real-world situations with technical difficulties. Residents were expected to ask the SP to adjust their position or the camera to fully visualize the face and the site of relevant symptoms and signs, especially the location of the sensory alteration, and to discuss an assessment and treatment plan.

The session ended when the resident and SP agreed on a plan or after 10 minutes elapsed. Trainees then received 5 minutes of in-person verbal feedback from the faculty observer. The SP and faculty observer completed written performance evaluations for each resident on communication skills and physical examination skills, respectively (eTables 2 and 3, links.lww.com/NE9/A40). The NYSIM provided timing announcements during the sessions: a 2-minute warning when the patient encounter was nearly complete, a 10-minute warning marking the end of the patient encounter, and a 5-minute prompt when faculty feedback was finished.

After completing a session consisting of 4 separate OSCEs, including the teleneurology OSCE, residents met with the faculty observers for a group debriefing session and completed a survey on their perceptions (eTable 4, links.lww.com/NE9/A40). The OSCEs were administered on 2 separate days to accommodate resident and faculty schedules.

##### Remote OSCE Design

Subsequently, we adapted the OSCE to be completely remote in the spring of 2021 and extended the teleneurology training sessions to second-year and third-year neurology trainees, even though they were already conducting teleneurology visits during the early part of the pandemic (Figure 1).

Several changes were needed to convert in-person OSCE elements to remote ones. A department faculty member played the SP instead of an actor. The OSCE creators spent 1 hour over Zoom training the SP and reviewing the same case-specific checklist that was used during the in-person simulation (eTable 2, links.lww.com/NE9/A40).

The SP, faculty observer, and second-year and third-year neurology trainees accessed the OSCE using their own devices at locations of their choosing. The faculty observer and the faculty member playing the SP used their office or their home. While the first-year trainees spent the afternoon in the simulation center and required coverage for 2 hours, the second-year and third-year residents needed coverage only for 15 minutes during their participation in the OSCE.

The OSCE script and time line were unchanged. The encounter remained 15 minutes long, including 5 minutes of verbal feedback. Instead of in-person verbal feedback, the faculty observer provided feedback virtually after the 10-minute patient encounter. Residents still received written feedback from the faculty observer and SP (eTable 3, links.lww.com/NE9/A40).

After conducting the first round of OSCEs in the simulation center, we decided that written faculty observer feedback should be structured and standardized. Working with the NYSIM colleagues, we created a standardized faculty feedback form for all future in-person and remote teleneurology OSCEs. Tasks were assessed as “not done,” “partly done,” or “well done,” accompanied by an area for free-text comments (eTable 3, links.lww.com/NE9/A40). There was no group debriefing session. Trainees filled out the post-OSCE survey online in Research Electronic Data Capture, a protected research management system (eTable 4).

The NYSIM did not provide support for our remote OSCEs. The faculty observer used a free online timer customized to provide prompts at the appropriate time points. The faculty observer hid the window during the remote OSCEs using the same method used for the in-person OSCEs.

##### Trainee OSCE Evaluations

In addition to the SP and faculty assessments, in a Post-Pre style evaluation survey, residents' self-reported change in interest in and comfort with performing teleneurology were assessed immediately after on-site and remote OSCEs.

#### Teleneurology Patient Exposure

Residents have been given opportunities to learn and practice teleneurology by incorporating teleneurology into resident continuity clinics and subspecialty rotations at multiple sites. Residents from all years of training have been performing telephone visits (audio-only) during continuity clinics at Bellevue Hospital. Second-year and third-year neurology residents performed audio/video visits at the Veterans Affairs New York Harbor Hospital continuity clinic. The residents attend continuity clinics once or twice per week throughout their training years. Slots were reserved for teleneurology visits at each location. The number of teleneurology visits varied depending on patient availability and preference.

Teleneurology has also been introduced into existing subspecialty rotations at the NYU Langone Health Faculty Group Practice. During these 2-week rotations, residents from all years of training attend multiple half-day clinics with attendings from different neurologic subspecialty services. Several faculty members designate half-days dedicated to virtual visits. Trainees participate in 1 or 2 half-day teleneurology clinics during their rotations.

### Standard Protocols, Approvals, Registrations, and Patient Consents

This educational program evaluation met NYU's criteria for certification as a quality improvement project rather than human subject research and was exempt from institutional review board review.

### Data Availability

Data not published within this article will be made available on request directed to the corresponding author.

## Results and Assessment Data

### Needs Assessment: Precurricular Survey

**Figure 2:**
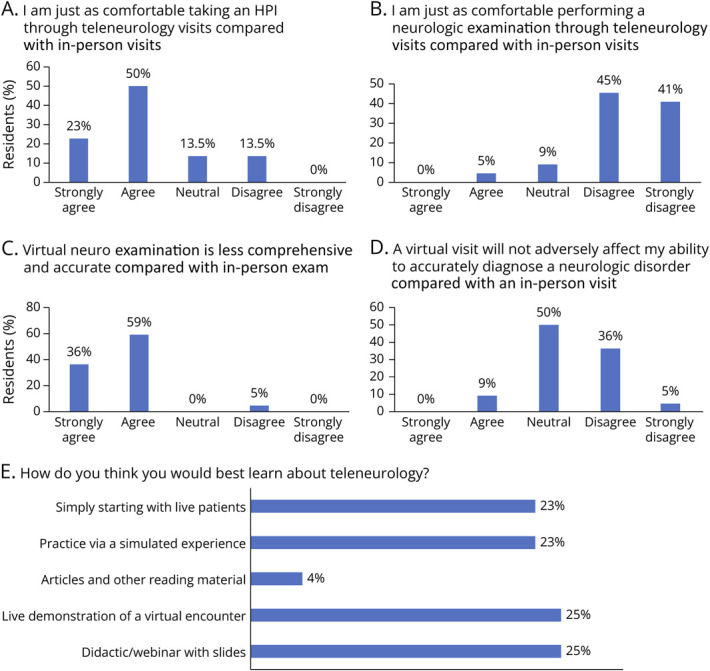
Major Findings From the Precurricular Survey of the Trainees This needs assessment was completed by the trainees anonymously prior to the start of the curriculum. Many trainees felt comfortable taking a history virtually but did not feel confident performing a neurologic examination virtually. The trainees felt that a variety of teaching modalities would be helpful in learning teleneurology. HPI = history of the present illness.

Twenty-two adult neurology, child neurology, and neuropsychiatry residents completed the precurricular survey. Most of the residents (73%) reported feeling comfortable taking a history of the present illness via teleneurology compared with in-person visits (Figure 2, A–D) but did not feel confident performing the neurologic examination (86%). Most residents (86%) desired more training on conducting effective teleneurology visits and thought that teleneurology should have an important role after the pandemic. Almost all trainees (95%) perceived the virtual neurologic examination to be less comprehensive and accurate compared with an in-person examination. Many (41%) were also concerned about their ability to accurately diagnose a neurologic disorder through teleneurology visits compared with in-person visits.

Residents reported that a variety of teaching modalities would help them learn about teleneurology: didactics with slides (25%), live demonstration of a virtual encounter (25%), simulated experience (23%), and directly starting with live patients (23%) (Figure 2E). Only a few of the residents (approximately 4%) chose articles or other reading material as their preferred method of learning. Of a total of 22 responders, 18 correctly chose 2 methods (as instructed) and 4 responders chose 3 methods. Therefore, 48 total responses were recorded.

Most free-text resident responses (73%) mentioned the need for further training in performing the virtual neurologic examination. Many trainees also expressed an interest in further telestroke training and more opportunities to perform teleneurology in outpatient settings.

### OSCE Participants

At the time this manuscript was prepared, the OSCE has been completed by a total of 68 trainees in 3 groups: 13 first-year neurology residents in the simulation center (Fall 2020), 38 second-year (N = 19) and third-year (N = 19) neurology residents remotely (Spring 2021), and 17 first-year neurology residents in the simulation center the following academic year (Fall 2021) (Figure 1). This included 54 adult neurology residents, 8 child neurology residents, and 6 neuropsychiatry residents.

#### On-site OSCE Trainee Feedback

**Figure 3:**
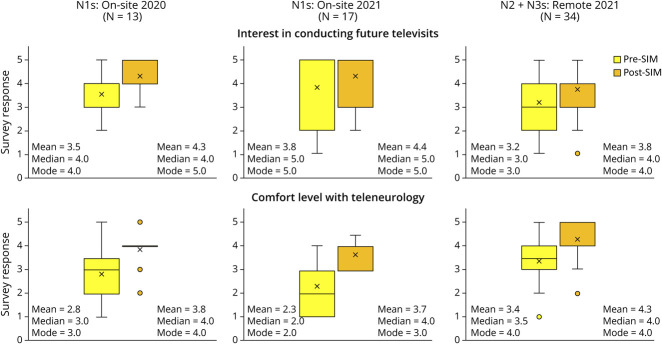
Trainees' Interest in and Comfort With Teleneurology Pre- and Postsimulation This comparison of interest in and comfort with teleneurology were reported by the trainees after the OSCE. Both levels of interest in and comfort with teleneurology increased postsimulation in on-site and remote settings.

First-year residents' interest in teleneurology increased after the simulation in 2020 and 2021 from a mean score of 3.5 to 4.3 (*p* < 0.05) and 3.8 to 4.4, respectively (Figure 3). The level of comfort with performing virtual visits after the simulation increased from a mean score of 2.8 to 3.8 (*p* < 0.05) and 2.3 to 3.7 (*p* < 0.05) (Figure 3). All first-year neurology trainees in 2020 and 2021, respectively, rated the OSCE as useful (4.8/5, 5/5) and educational (4.8/5, 5/5), and all thought the teleneurology OSCE should be repeated in the future (100%, 100%) (Figure 3). Residents identified performing the virtual neurologic examination and time constraints as the most challenging aspects of the simulation.

#### On-site OSCE SP Evaluations

**Figure 4:**
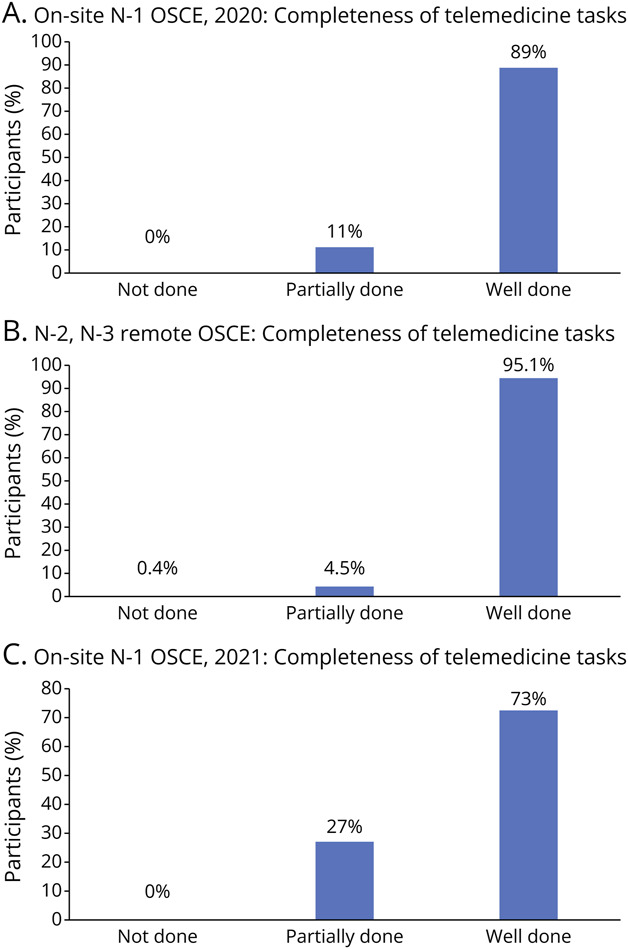
Trainee Performance on Completing Telemedicine Tasks During the OSCE Trainee performance on completing telemedicine tasks was assessed by the SP and categorized into “not done”, “partially done” or “well done” based on a predetermined criteria. Most of the trainees scored “well done” or “partially done” on the telemedicine tasks. OSCE = objective structured clinical examination; SP = standardized patient.

The SP's written feedback is presented in Figure 4, A and C as well as summarized in eTable 5 (links.lww.com/NE9/A40). Notably, the residents had good “webside” manner. The N1 residents in 2020 on average completed 89% of the telemedicine skills/tasks as well done and 11% of these tasks as partially done. The N1 residents in 2021 on average completed 73% of the telemedicine skills/tasks as well done and 27% of these tasks as partially done.

#### On-site Faculty Observer Evaluations

**Figure 5:**
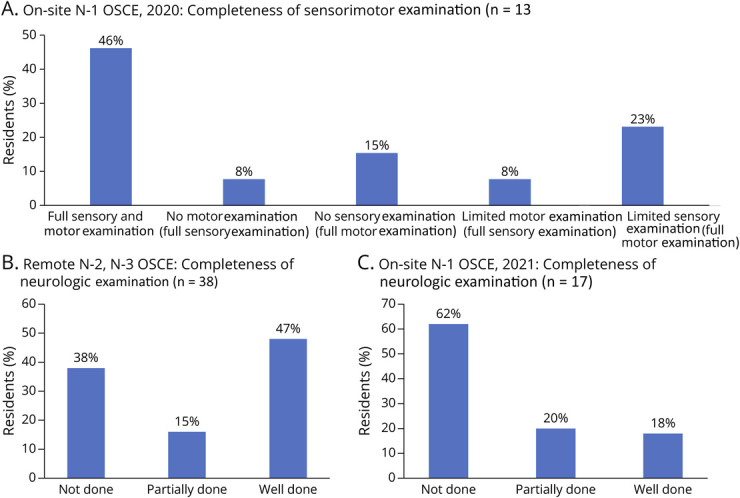
Trainee Performance on Completing Examination During the OSCE Trainee performance on completing telemedicine tasks was assessed by the faculty observer and categorized into “not done”, “partially done”, or “well done” based on a predetermined criteria. Many components of the neurologic exam were “not done” or “partially done”. OSCE = objective structured clinical examination.

For the first-year residents in 2020, the faculty observer reported that more than a third (38%) of the residents had difficulty with optimizing the SP's camera to fully visualize the patient and a little more than half of the residents (54%) omitted or partially performed the sensory or motor examinations during the encounter (Figure 5A). In addition, approximately half of our residents did not suggest the correct diagnosis.

For the first-year residents in 2021, the faculty observer reported that approximately 18% of the virtual neurologic examinations were well done, 20% were partially done, and 62% were not done (Figure 5C).

#### Remote OSCE Trainee Feedback

When the second-year and third-year neurology residents participated in the same OSCE remotely, residents' (N = 38) interest in teleneurology increased from a mean score of 3.2 to 3.8 (*p* < 0.05) after the simulation (Figure 3). The level of comfort with performing virtual visits increased from a mean score of 3.4 to 4.3 (*p* < 0.05) after the simulation. Trainees rated the OSCE as useful (4.3/5) and educational (4.3/5), and 88% (33/38) thought the teleneurology OSCE should be repeated in the future, 12% said “maybe,” and 0% said “no.” Residents identified performing the virtual neurologic examination and time constraints as the most challenging aspects of the OSCE.

#### Remote OSCE SP Evaluations

The SP's written feedback is presented in Figure 4B and summarized in eTable 5 (links.lww.com/NE9/A40). Notably, the residents had good “webside” manner, on average completing 95.1% of the telemedicine skills/tasks as well done, 4.5% of these tasks as partially done, and 0.3% of these tasks as not done.

#### Remote OSCE Faculty Observer Evaluations

The faculty observer reported that approximately 47% of the virtual neurologic examinations were well done, 15% were partially done, and 38% were not done (Figure 5B). Reflexes and some of the cranial nerve examinations were the elements most frequently not performed.

#### Free-text OSCE Feedback From On-site and Remote Residents

Trainees from all levels reported similar challenges and suggestions for improving the OSCE (Table 1 and eTable 6, links.lww.com/NE9/A40). Regardless of whether the first-year, second-year, and third-year neurology trainees completed the OSCE in the simulation center or remotely, residents emphasized that the teleneurology examination skills and technical challenges, such as camera positioning, were the most difficult portions of the teleneurology OSCE. Residents requested opportunities “to practice the examination more,” “perhaps something involving cranial nerves,” or “multiple patients with some motor or cerebellar signs” (eTable 6).

**Table 1:**
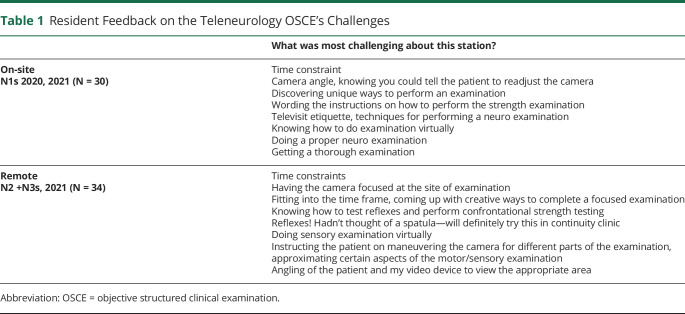
Resident Feedback on the Teleneurology OSCE's Challenges

## Discussion and Lessons Learned

We found residents preferred active rather than passive teleneurology training from their needs assessment, favoring modalities requiring a degree of participation from learners. Telehealth curricula have been implemented in other programs, although few have capitalized on the preference for active learning by way of simulation technology. One neurology program developed an experiential outpatient teleneurology curriculum involving online modules followed by clinic experiences.^9^ In 2020, an internal medicine residency program introduced didactics, a resource guide, and real-time exposure for their trainees.^30^ One program incorporated simulation to teach telestroke.^12^

Our teleneurology curriculum incorporates multimodal learning, which includes reading material, didactics, live demonstration, and simulated as well as real-life clinical experience throughout all years of training in various environments. Simulation allows participants to increase exposure to simulated real-life conditions and receive real-time feedback. Participation in neurology-focused and non-neurology–focused simulations has been linked to improvements in patient care.^31-33^

While training programs outside of neurology have started to use simulation to teach telemedicine, these often focus on fostering telemedicine communication skills.^28,29,34,35^ Our simulation focuses on teaching and reinforcing neurologic examination skills, an essential component of clinical neurology. In-person OSCEs have been effective educational tools across a spectrum of clinical scenarios in neurology.^36-42^ Simulation has the potential to enable trainees to adapt and strengthen the remote neurologic examination and communication skills, essential components of delivering high-quality health care with positive medical outcomes.^43,44^

We learned several lessons designing the teleneurology OSCE. Given the difficulty many residents had with the teleneurology examination, a case with a sensory deficit was a good initial experience. Anecdotally, the remote sensory examination is more straightforward than remote motor, reflex, or cranial nerve testing. It is easier for an SP to simulate a sensory deficit compared with other neurologic examination findings, other than, perhaps, mental status testing. Allowing learners the opportunity to transfer their in-person examination skills to a virtual environment was well received. Performing the virtual neurologic examination more expertly is an area that residents identified as challenging in the needs assessment. In addition to a lack of familiarity and expertise in the virtual neurologic examination, the time constraint of the OSCE (which was reported by the trainees) may have contributed to partially or not completing certain components of the neurologic examination. This suggests focusing on teaching trainees to perform the virtual neurologic examination more efficiently. Intentionally having the SP's face only partially visualized at the beginning of the encounter was a good way to test real-world suboptimal technical experiences. Technical skills involved in virtual encounters need to be acknowledged and trainees should be exposed to environments where they can practice these skills in real time.

Residents overwhelmingly asked for more simulations to practice and implement new and more advanced remote examination skills. We have subsequently incorporated teleneurology principles into 2 more of our simulated scenarios, one in which a diagnosis of nonepileptic events is provided to an SP previously in-person but now via a telemedicine encounter and another in which trainees perform the NIHSS on an SP via telestroke encounter.^39,45^

We learned that the OSCE could be performed remotely without the use of a physical simulation center. Based on the discussion with the facilitators and the developers of the OSCE, we learned that there are pros and cons to remote vs in-person simulations (Table 2). Conducting remote simulations allows for logistical flexibility. This may incur less administrative burden to organize because residents' participation can occur from any private location (such as a call room) using a mobile electronic device such as a cell phone. Remote OSCEs minimize scheduling conflicts because trainees do not need to be excused from rotations for prolonged periods requiring cross-coverage. Performing the teleneurology OSCE remotely from a location that is not a simulation center is more representative of real-world experience.

**Table 2:**
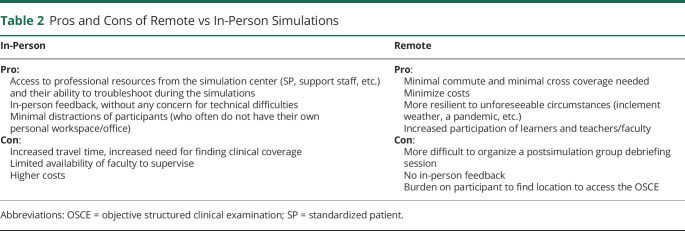
Pros and Cons of Remote vs In-Person Simulations

Our fully remote simulation did not have a group debriefing session afterward. A potential solution may involve a group debriefing session using a teleconference platform. A physician portrayed the SP, instead of an actor, minimizing cost and the time required to train the faculty member, given the medical background. On the contrary, SPs are trained to standardize their clinical presentation and evaluation, training that faculty do not typically receive. Many simulation centers may provide resources, SPs, and assistance even during remote simulations. Expanding the use of remote simulations may especially enhance trainee education in programs that do not have access to a simulation center or have limited finances to support simulations.

Our experience demonstrates that a remote OSCE is feasible. We successfully implemented a teleneurology simulation with and without the use of a simulation center. While its effectiveness as an educational tool requires further investigation, feedback from trainees regarding both the in-person and remote OSCEs demonstrated an increase in residents' interest and confidence in performing virtual visits after participating.

With the feedback from a supervised virtual encounter such as the OSCE and continued opportunities for supervised clinical encounters, residents will be able to develop teleneurology skills as they progress through their training. This will likely become an essential skill set if, as predicted, teleneurology remains mainstream in the post-COVID era.^46-48^

Our curriculum has components that can be incorporated into existing residency curricula in other institutions. Reading material, didactics, and live demonstrations can often be offered without significant cost to educational programs. Additional costs may include setting up the infrastructure for video/audio communication system if not already in place, training SPs for teleneurology OSCEs, and having faculty observers available for the sessions. A part of the resident clinic schedule can be dedicated to teleneurology patients for real-world experience.

Our project has limitations. Our simulation addresses only a subset of the many potential technical challenges seen in the practice of teleneurology and does not accurately represent the wide range of patient technologic literacy. The assessment the faculty observer completed for the first cohort of learners was not standardized. We plan to ensure uniformity, so all learners are assessed by the same metrics and checklists. We did not survey residents immediately before they participated in the simulation. Because the SPs were different between the remote and on-site OSCEs, there is a potential for inter-rater variability in reporting of the telemedicine-specific tasks. From a reproducibility and scalability perspective, some residency programs may not have access to a simulation center or faculty willing to participate in an in-person or remote simulation. We recognize that further studies need to be conducted to validate our assessment tools. We hope to test the validity of our assessment tools, including performing inter-rater reliability and retest reliability testing with future iterations of the OSCE. We also recognize that there has not been a reiteration of remote teleneurology OSCE because the OSCE was fully incorporated into the in-person 4-station OSCE on core competencies for first-year trainees.

There are additional opportunities to further develop our teleneurology curriculum to enhance trainees' exposure, skills, and competency evaluation. As the teleneurology evidence base grows, we plan to incorporate new information into our curriculum. We recently identified the 14 elements of the remote neurologic examination that our faculty members found were most useful for medical decision-making during virtual encounters.^49^ Uploading videos of sample encounters to our central resident website for review may be a valuable educational resource. We are also considering adding dedicated teleneurology elective rotations. We could institute formalized assessment and evaluation tools during these clinical exposures, including those for the resident continuity clinics and subspecialty clinics.

Other future directions include creating additional simulations with diverse clinical scenarios including some thought to be less amenable to remote evaluation. There are opportunities for expanding access to teleneurology simulations for additional learners, including faculty members and medical students.^50^ A repository of simulations that can be shared across institutions could enhance the breadth and depth of neurology education training. A particularly important step would be to measure whether this type of teaching method changes trainee behavior and improves patient outcomes. Assessing the clinical documentation of telemedicine encounters could also be a way to capture the impact of the curriculum. Learning to effectively perform telemedicine with the help of caregivers for patients who are unable to participate in the virtual encounter on their own may also be an important skill to learn.

Current clinical evaluations to ensure competency before graduation include the American Board of Psychiatry and Neurology Neurology Clinical Evaluation Exercise. However, there are no standardized processes to ensure teleneurology clinical competency. Teleneurology simulations could assess trainee's skill acquisition and help track the effectiveness of the teleneurology simulation. Creating requisite simulations that feature complex conditions within general neurology and across its subspecialties may allow trainees to practice and cultivate more advanced examination techniques.

We believe that teleneurology will be a commonly accepted and widespread modality of patient care in the future. Robust teleneurology education should become an essential part of residency training to prepare residents to provide high-quality virtual patient care after training.

## Glossary

COVID-19: coronavirus disease 2019
NIHSS: NIH Stroke Scale
NYSIM: New York Simulation Center
NYU: New York University
NYUGSOM: NYU Grossman School of Medicine
OSCE: objective structured clinical examination
SP: standardized patient
